# Investigation of smoke-taint precursor modification by glycosidase activity in diverse wine yeast and bacterial strains

**DOI:** 10.3389/fmicb.2025.1679638

**Published:** 2025-10-31

**Authors:** Hailan Piao, Thomas S. Collins, Thomas Henick-Kling

**Affiliations:** Department of Viticulture and Enology, Washington State University, Richland, WA, United States

**Keywords:** glycosidase, smoke-taint, yeast and bacteria, wine aroma, flavor extract, glycoside precursor

## Abstract

The increasing prevalence of wildfires presents a growing risk to wine production through the development of smoke-taint, a sensory defect in wine caused by volatile phenols absorbed by grapevines during smoke exposure. In grapes and wine, these volatile phenols are often present in glycosylated forms that can be hydrolyzed during fermentation, releasing undesirable smoky aromas. This study investigated the glycosidase activity of diverse *Saccharomyces cerevisiae* and *Oenococcus oeni* strains to evaluate their roles in modulating smoke-taint-associated glycosides during wine fermentation. Mini-scale alcoholic and malolactic fermentations were conducted in synthetic media enriched with flavor extracts from smoke-exposed grapes using reverse osmosis. LC–MS profiling revealed strain-dependent hydrolysis of glycosides, linked to smoke-taint. Notably, *S. cerevisiae* strains UCD514 and UCD525, and *O. oeni* strain UCD199, showed the highest glycosidase activity. Principal component analysis further confirmed that individual microbial strains had distinct metabolic impacts on glycoside profiles. This study highlights a wide range of glycosides that can be hydrolyzed by wine yeast and bacteria. These findings demonstrate the dual potential of microbial glycosidase activity to mitigate smoke-taint while enhancing wine aroma. In addition, the results help distinguish smoke-taint-associated glycosides that are resistant to microbial hydrolysis from those that are readily cleaved, enabling targeted removal of released aroma compounds through downstream filtration approaches.

## Introduction

The global wine industry is significantly threatened by the increasing frequency and severity of wildfires, a trend closely linked to climate change (van Leeuwen et al., 2024; Sun et al., 2023). Wildfires pose multifaceted risks to winemaking, with smoke-taint being among the most impactful consequences, both sensorially and economically. Smoke exposure of grapevines and fruit can lead to significantly degraded wine quality through the development of objectionable smoke-taint aromas (Hayasaka et al., 2010a,b). When grapevines are exposed to smoke, volatile phenols absorbed through the grape skins and leaves are then bound to sugar molecules and transformed into glycosides. During fermentation and wine aging, these bound compounds are hydrolyzed, releasing undesirable smoky flavors to the wine (Szeto et al., 2020; Culbert et al., 2021; Whitmore et al., 2021). The presence of smoke-taint can result in devastating financial losses for wine producers, due to diminished product quality and even unmarketable wines (Summerson et al., 2021).

Smoke-taint manifests in wines as undesirable sensory attributes, often characterized by off-notes such as “smoky,” “dirty,” “smoked meat,” “earthy,” and “burnt” descriptors (Kennison et al., 2008; Kennison et al., 2007). These off-aroma and flavors severely negatively impact consumer acceptance and brand reputation. At the chemical level, smoke-taint is primarily caused by volatile phenols, including guaiacol, o-cresol, m-cresol, 4-methylguaiacol, 4-ethylguaiacol, and 4-ethylphenol (Summerson et al., 2021; Yang et al., 2021; Whitmore et al., 2021; Kennison et al., 2008). These compounds are often found in glycosylated metabolites, non-volatile form within the grape tissue, rendering them initially flavorless until released during fermentation (Kennison et al., 2008; Parker et al., 2012; Hayasaka et al., 2013; Hayasaka et al., 2010b). This glycosylation acts as a reservoir for aroma precursors that can be cleaved by microbial enzymic activity. During fermentation and wine aging, enzymatic hydrolysis can release these volatile phenols, resulting in the sensory perception of smoke-taint in the finished product (Cui et al., 2024; Noestheden et al., 2018; Parker et al., 2012). The critical contribution of volatile sulfur compounds to the perception of smoke taint in wine was identified by Tomasino et al. (2023).

Recent research has increasingly focused on identifying and refining mitigation strategies during winemaking and post-exposure treatments aimed at reducing or masking the sensory impact of smoke-taint, an off-flavor condition caused by wildfire smoke exposure in grapes (Oberholster et al., 2022). Despite numerous approaches that have been explored, including activated carbon fining, reverse osmosis, and enzymatic treatments, a standardized and universally effective solution for smoke-taint management remains elusive. Given the wine industry’s critical reliance on nuanced sensory attributes and increasing incidence of wildfires, developing reliable and scalable mitigation strategies is an urgent research priority.

The glycosidic activity of *S. cerevisiae* yeasts has been widely studied using standardized glycosides such as p-NPG (p-nitrophenol-beta-D-glucopyranoside), 4-MUG (4-methylumbelliferyl-*β*-D-glucose), arbutin (hydroquinone β-D-glucopyranoside), and esculin (esculetin 6-β-D-glucoside) in model solutions. Studies have also examined single-strain fermentations of *S. cerevisiae* in model solutions containing grape glycosides, and finally in fermentations with natural grape must (Zhang et al., 2021; Hernández et al., 2003; Spagna et al., 2002). Although tests in model solutions often indicated low glycosidic activity among *S. cerevisiae*, many studies using grape glycosides have demonstrated widespread and diverse glycosidic activity in this species (Ugliano et al., 2006; Ugliano et al., 2007; Caffrey and Ebeler, 2021; Zhang et al., 2021). The study by Ugliano et al. (2006) showed that *S. cerevisiae* demonstrates activity against grape mono- and disaccharide glycosides, and concluded that the extent of the hydrolysis depends on the chemical structure of the aglycone. Therefore, results obtained with p-NPG alone cannot fully reflect the activity of a yeast against the diverse glycosides present in grapes.

This study investigates which smoke-taint-associated glycosides in grape must can be hydrolyzed by various wine yeasts and bacteria, leading to the release of free aroma compounds. Glycosidic activity was examined in a diverse selection of wine yeasts and malolactic bacteria, both of which are known to harbor glycosidase enzymes that catalyze the hydrolysis of glycosidic bonds. These enzymes play a dual role: enhancing wine aroma by liberating desirable volatile compounds from glycosylated precursors, or exacerbating smoke-taint by releasing latent volatile phenols (Graf et al., 2022; Belda et al., 2016; Michlmayr et al., 2010).

In this study, we focused on the primary wine yeast and lactic acid bacterium, *S. cerevisiae* and *O. oeni*. We aimed to explore the extent of glycosidase activity among diverse strains of these microorganisms against smoke-taint-associated glycosides. Specifically, 10 *S. cerevisiae* strains and 5 *O. oeni* strains were assessed for their ability to hydrolyze mono- and di-glycosides of hexoses and pentoses. This biological approach provides winemakers with valuable insight into the potential of wine yeasts and bacteria to mitigate the release of smoke-derived volatile phenols during alcoholic and malolactic fermentation.

## Materials and methods

### Strains and growth conditions

To investigate the diversity of glycosidic activity in *S. cerevisiae* and *O. oeni,* we selected strains from the University of California, Davis strain collection, isolated from diverse grape and wine environments, including different countries and grape must types (Supplementary Table S1). Yeast cultures were inoculated into WL Nutrition Medium (HiMedia), an enriched medium optimized for the cultivation of wine yeast. Lactic acid bacteria strains were grown in Lactobacillus MRS broth (BD Difco) supplemented with 0.5% (w/v) fructose and 0.2% (w/v) L-malic acid to mimic wine-like conditions and support malolactic activity. The medium pH was adjusted to 3.5 with tartaric acid. Cultures were incubated under standard growth conditions: 30 °C for yeast and 25 °C for bacteria.

### Preparation of grape flavor extract

Merlot grapes (*Vitis vinifera L.*) were exposed to smoke under controlled conditions. A glycoside-enriched flavor extract (permeate) was prepared from the juice of smoke-exposed grapes using reverse osmosis filtration. The juice was processed through a reverse osmosis membrane with a 1,000 Da molecular weight cutoff, which selectively retained high-molecular-weight compounds while permitting small molecules to pass. The resulting permeate was collected and stored at −20 °C for subsequent analyses.

### Preparation of synthetic grape juice and wine media

The synthetic base medium was prepared by modifying a previously described synthetic grape must (SGM) formulation (Viana et al., 2014). Briefly, 3.40 g/L yeast nitrogen base (YNB) without amino acids and ammonium sulfate (MP Biomedicals), 3.85 g/L essential amino acids (MP Biomedicals), 3.0 g/L tartaric acid (RPI), and 2.0 g/L L-malic acid (ACROS Organic) were dissolved in distilled water. For the preparation of synthetic grape juice, 100 g/L each of glucose (VWR BDH Chemicals) and fructose (VWR Life Science) was added to the base medium. To simulate synthetic wine, 12% (v/v) ethanol was included. The pH of all media was adjusted to 3.5 with potassium bicarbonate, followed by sterile filtration through a 0.22 μm membrane. A 20% (v/v) aliquot of the prepared flavor extract was subsequently added to each formulation. Detailed compositions of the synthetic juice and wine media are listed in Supplementary Table S2.

### Mini-scale alcoholic fermentation

Mini-scale alcoholic fermentations were conducted using synthetic grape juice supplemented with a glycoside-rich flavor extract (Supplementary Table S2) to evaluate yeast-driven glycosidase activity on the smoke-taint-related glycoside precursors. A total of 4.5 L of supplemented synthetic juice (containing 20% (v/v) flavor extract) was aliquoted into sterilized 125 mL Erlenmeyer glass flasks, which were sealed with aluminum foil and parafilm. Each flask was inoculated with pre-cultured *S. cerevisiae* strains at an initial cell density of 1 × 10^7^ cells/mL. Fermentations were conducted at 20 °C with gentle agitation on an orbital shaker, and residual sugar was periodically monitored using enzymatic analysis (Admeo Y15, Napa, CA) to determine fermentation progress and completion. Three uninoculated flasks were prepared under identical conditions to serve as negative controls, labeled “No yeast.” All fermentations were carried out in triplicate. Final samples collected at the end of incubation were immediately frozen and stored at −20 °C for subsequent glycoside analysis.

### Mini-scale malolactic fermentation

Mini-scale malolactic fermentations were performed using synthetic wine supplemented with a glycoside-rich flavor extract (Supplementary Table S2) to evaluate bacteria-driven glycosidase activity on smoke-taint-related glycoside precursors. A total of 2 L of synthetic wine supplemented with 20% (v/v) flavor extract was distributed into sterilized 50 mL Erlenmeyer glass flasks. Each flask was inoculated with pre-cultured *O. oeni* strains at an initial cell density of 1 × 10^8^ cells/mL. Fermentations were carried out at 25 °C under static conditions. L-malic acid concentrations were monitored at regular intervals using enzymatic analysis (Admeo, Y15, Napa, CA) to assess the progress of malolactic fermentation. Upon completion, all samples were frozen at −20 °C for subsequent glycoside analysis. All fermentations were carried out in triplicate, and three uninoculated flasks prepared under identical conditions served as negative controls, labeled “No MLB” (Malolactic Bacteria).

### HPLC sample preparation and analysis conditions

For each individual strain (yeast and bacteria) and the corresponding control samples, a total of 9 samples (3 independent biological replicates, each prepared in triplicate as technical repeats) were analyzed using an Agilent 1,290 Infinity ultra-high-pressure liquid chromatography (UHLPC) system coupled to an Agilent G6545A quadrupole time-of-flight (QTOF) mass spectrometer (Agilent Technologies, Santa Clara, CA).

Samples (1.5 mL) were first filtered through a 0.45 μm syringe filter to remove particulate matter and then transferred into HPLC vials for analysis. Chromatography and QTOF operating conditions followed the laboratory methods described by Tomasino et al. (2023). Briefly, 5 μL injections were made for each sample, and separations were performed using an Agilent Zorbax Eclipse Plus C18 column. A reversed-phase separation employed 0.1% acetic acid in water (phase A) and 0.1% acetic acid in methanol (phase B). The gradient started at 97% A/3% B, shifted linearly to 80% B at 11.0 min, and then to 100% B at 14 min. The concentration was maintained at 100% B for 1.0 min before returning to the initial 97% A/3% B at 16.0 min. The total run time was 17.0 min at a constant flow rate of 0.7 mL/min.

For the QTOF analysis, an Agilent Dual Jet stream electrospray ionization (ESI) source was used in negative mode. The instrument was calibrated according to the manufacturer’s instructions prior to analysis. Mass data were acquired in both profile and centroid modes over a range of 100–1,100 m/z with a scan rate of 3 spectra/s. Source parameters were identical to those described in Tomasino et al. (2023).

### Data analysis: untargeted methods

Raw LC–MS data (.d format) were processed using MassHunter Qualitative Analysis Software B.10.0 (Agilent Technologies, Santa Clara, CA). The Molecular Feature Extraction (MFE) algorithm was applied to identify compounds based on common ion species such as [M + H]^+^, [M-H]^-^, and [M + CH3COO]^−^ as well as on characteristic neutral losses associated with glycosides. Screening for these neutral losses enabled the detection of specific moieties within the analyzed compounds. The workflow and MFE parameters followed Agilent’s QTOF recommendations. A total of 99 datasets from yeast fermentations and 54 from malolactic fermentations were analyzed.

### Compound extraction and alignment using MassHunter profiler profession

Molecular futures detected by MFE were exported as Compound Exchange Format (.cef) files and processed using Agilent MassHunter Profiler Professional (MPP) software. A new project was created with two separate experiments: one for yeast alcoholic fermentation data and one for bacterial malolactic fermentation data. Since compounds were identified solely based on molecular features (neutral mass and retention time), the experiment type was set to “unidentified.” Datasets that generated processing errors in MPP were excluded, and only quality-controlled (“clean”) datasets were retained for downstream analysis. For compound extraction, an abundance threshold of 5,000, based on the integrated chromatographic peak area, was applied.

### Glycoside compound detection

All extracted compounds were evaluated individually across replicates using automated Excel functions and an in-house glycoside database containing approximately 2,000 potential smoke-taint glycoside compounds. Accurate mass matches were applied to identify potential smoke-taint glycosides. For each replicate, a “+” was assigned if the compound was detected in at least one replicate of a given sample (yeast, bacterial strains, or control), indicating that the compound was present and not hydrolyzed. If a compound was consistently absent across all replicates of a sample, it was recorded as “-,” indicating that it was not present and therefore hydrolyzed. Compounds detected in yeast or bacterial samples but absent in the corresponding control samples (“No yeast” or “No MLB”) were not considered.

### Principal component analysis

Principal component analysis (PCA) was performed using the *prcomp* function in R (version 4.2.2). Plots were generated with the ggplot2 package (Wickham, 2009), and 95% confidence ellipses for each group were visualized using the stat_ellipse function. The PCA was conducted using all smoke-taint glycosides identified from the in-house putative smoke-taint compound database, with the complete list of identified compounds provided in Supplementary Tables S4, S6.

## Results

### Yeast alcoholic fermentation in synthetic grape juice

To identify the potential glycosidase activity among diverse *S. cerevisiae* strains, an initial screening was conducted to evaluate their ability to ferment (data not shown) in a synthetic grape juice medium formulated to mimic the natural grape must matrix (composition provided in Supplementary Table S2). Based on this preliminary assessment, 10 *S. cerevisiae* strains that fermented efficiently and represented a broad range of ecological and geographical origins relevant to winemaking were selected for detailed investigation (Supplementary Table S1). This set included strains derived from both commercial and native fermentation environments, enabling a comparative analysis of strain-specific fermentation performance and glycosidase activity.

**Figure 1:**
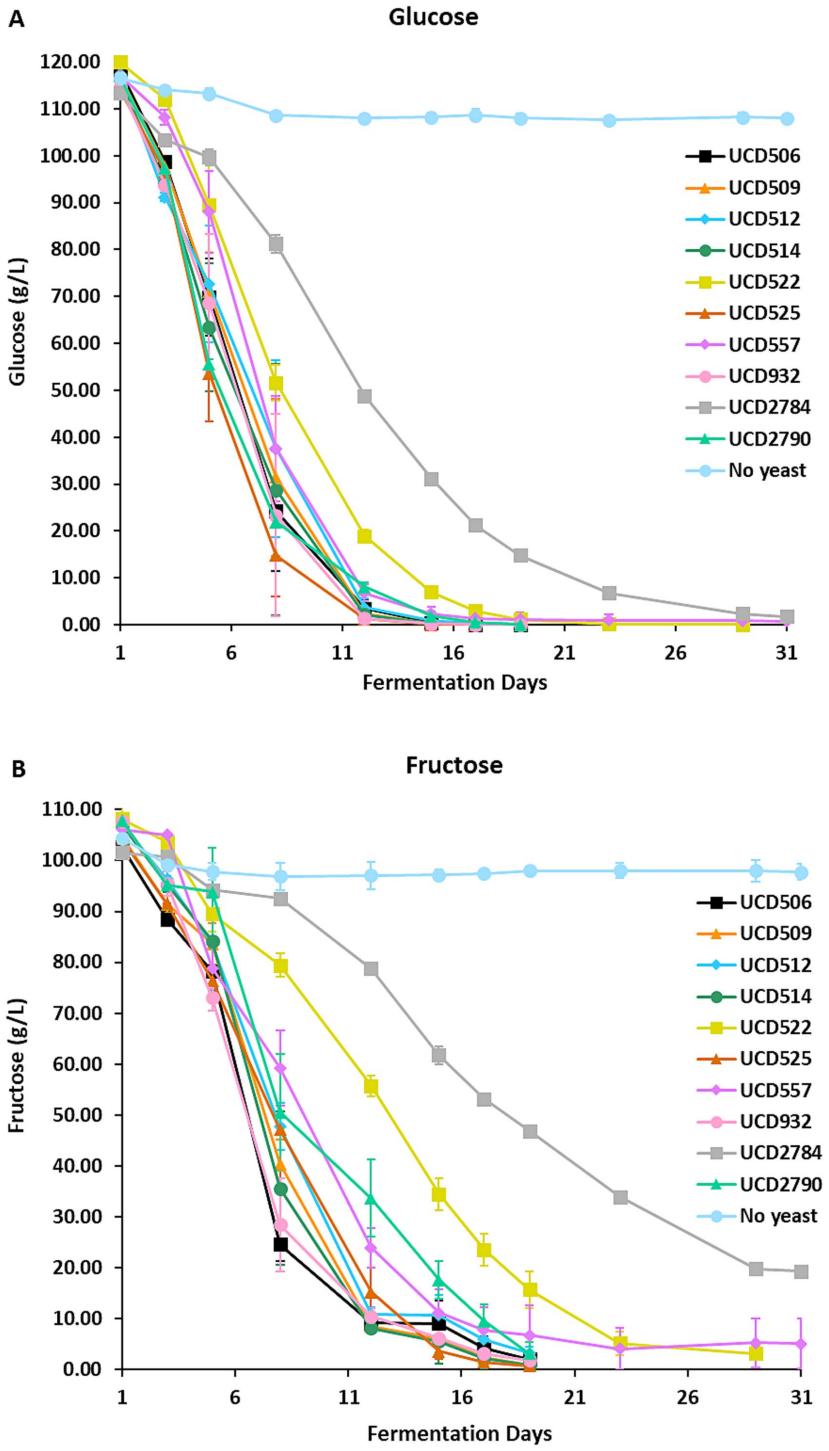
Alcoholic fermentation in the synthetic juice. Glucose **(A)** and fructose **(B)** concentrations were measured over 31 days of fermentation using 10 different *S. cerevisiae* strains and a “No yeast” control. Data points represent the mean of three independent biological replicates, and error bars indicate standard deviations.

All 10 yeast strains actively metabolized sugars, as evidenced by a progressive reduction in glucose and fructose concentrations throughout fermentation (Figure 1). Most strains completed fermentation within 19 days, demonstrating efficient fermentation kinetics under the experimental conditions. However, distinct variations in fermentation dynamics were observed. Strains UCD522 and UCD557 required approximately 10 additional days to achieve near-complete sugar utilization, indicating slower metabolic rates compared to the other strains. In contrast, strain UCD2784 exhibited sluggish or incomplete fermentation, retaining ~18% residual fructose after 31 days, suggesting limited metabolic capacity or possible stress responses affecting sugar metabolism.

The non-inoculated “No yeast” control exhibited only slight reductions in glucose (~8 g/L) and fructose (~ 7 g/L) throughout the incubation period (Figures 1A,B). As the synthetic medium was filter-sterilized, no microbial fermentation occurred; thus, these minor changes in sugar concentration likely reflect non-enzymatic chemical processes (e.g., slow oxidation, interactions with medium components, or binding to flavor extracts) rather than active microbial sugar consumption. The absence of microbial metabolic activity in the “No yeast” control supports that the changes observed in the inoculated fermentations were attributable to yeast metabolism. Variability in sugar utilization among strains highlights strain-dependent differences in fermentation efficiency and potentially in glycosidase expression profiles (Qin et al., 2021). Strains that metabolize sugars more efficiently may exhibit higher or earlier glycosidase activity, whereas those with delayed or incomplete sugar utilization may release glycosidically bound smoke-taint compounds more slowly or incompletely.

### Bacteria malolactic fermentation in synthetic wine

To evaluate glycosidase activity among *O. oeni* strains, a preliminary screening was performed to assess their capacity for malolactic fermentation (MLF) (data not shown) in a synthetic wine medium. MLF progress was monitored by quantifying L-malic acid concentrations with an enzymatic assay (Admeo, Inc.). Based on MLF efficiency and the diverse geographic and ecological origins of strains, 5 *O. oeni* strains were selected for further investigation (Supplementary Table S1).

**Figure 2:**
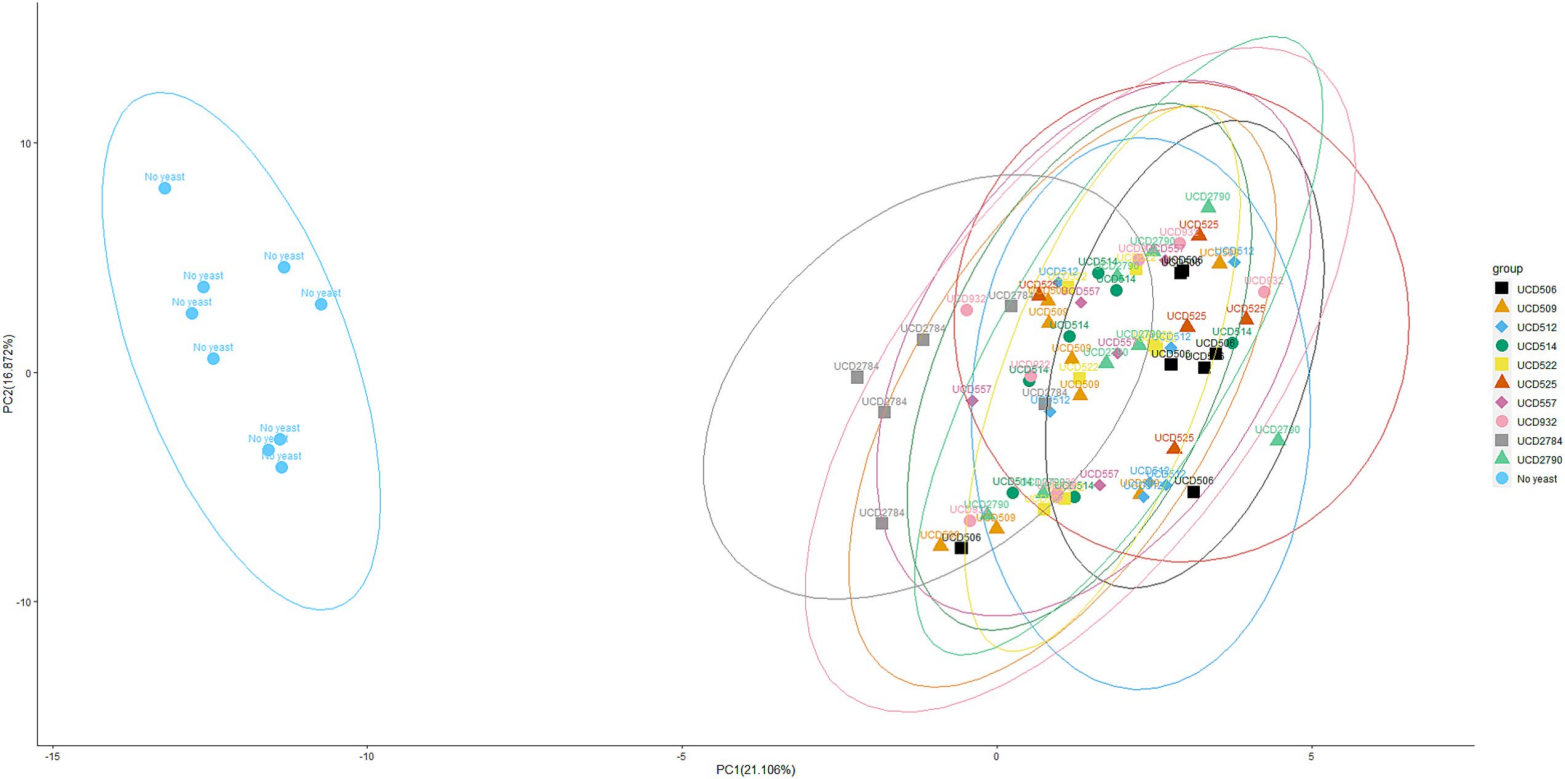
Principal component analysis (PCA) of glycoside profiles from mini-scale alcoholic fermentation. PCA plot showing the distribution of glycoside profiles obtained after fermentation with 10 different *S. cerevisiae* strains and a “No yeast” control. Each point represents both biological and technical replicates. Each point represents both biological and technical replicates. For each strain, 9 samples were analyzed in total: 3 independent biological replicates, each prepared in triplicate as technical repeats, to minimize variability from HPLC instrument runs. Datasets producing inconsistent results were excluded, and only “clean” data were retained for PCA analysis. Data are projected onto the first two principal components, with the percentage of total variance explained by each component indicated on the respective axes. Ellipses represent the 95% confidence interval around the multivariate mean of each strain, providing a visual estimate of clustering and variability.

Mini-scale malolactic fermentations were then carried out in the same synthetic wine (composition provided in Supplementary Table S2) to assess each strain’s potential for glycosidase-mediated transformation of smoke-tainted precursor compounds (Figure 2). All 5 strains demonstrated effective MLF activity, although differences in the kinetics of malic acid conversion were observed. Strains UCD167, UCD224, and UCD199 achieved 83–88% reduction in L-malic acid within the first 7 days, indicating rapid initiation of MLF. By day 21, all strains had completed MLF, converting 90–98% of L-malic acid to L-lactic acid. In contrast, the uninoculated “No MLB” control retained its initial L-malic acid concentration throughout the incubation period, confirming the absence of microbial activity.

The high MLF activity of the selected *O. oeni* strains emphasizes their robust metabolic capacity and their potential role in modulating glycosidic precursors associated with smoke-taint during wine fermentation. Strain-dependent differences in the rate of malic acid conversion suggest that certain strains may facilitate earlier or more extensive release of smoke-taint compounds from glycosidically bound precursors.

### Glycoside hydrolysis by yeast

Untargeted metabolite profiling was performed on 99 samples from yeast fermentation, representing 10 yeast strains and one uninoculated control. Each fermentation was conducted with three biological repeats, and each of the biological samples was prepared in three technical repeats for the HPLC analysis. Qualitative and quantitative LC–MS analyses identified approximately 1,550 compounds across all fermentations and control samples.

To specifically identify smoke-taint-related glycosidic metabolites, an in-house database of potential smoke-taint glycoside accurate masses was applied, resulting in the detection of 93 putative glycoside compounds, which were used to evaluate enzymatic hydrolysis across all yeast samples (Supplementary Table S4). Of these compounds, 62 were not hydrolyzed by any of the tested yeast strains, indicating that they were resistant to both enzymatic and chemical hydrolysis. Among the 10 tested *S. cerevisiae* strains, UCD514 and UCD525 hydrolyzed the highest number of glycosides, each hydrolyzing 21 out of 93 compounds (Supplementary Table S3). The remaining strains demonstrated varying hydrolysis patterns, highlighting strain-dependent differences in glycosidase activity (Table 1).

**Table 1:** Hydrolysis of smoke-taint-related glycoside compounds by yeast strains.

	UCD506	UCD509	UCD512	UCD514	UCD522	UCD525	UCD557	UCD932	UCD2784	UCD2790	No yeast
Compounds	Smoke-taint glycosides										
4-ethylguaiacol hexonate	+	+	−	−	+	−	+	−	−	−	+
4-ethylguaiacol hexosylhexoside	+	−	+	+	+	+	+	+	−	+	+
Guaiacol caftaroylpentoside	−	−	+	−	+	+	−	+	−	+	+
Guaiacol galloylpentoside	−	−	−	−	−	−	−	−	−	−	+
Guaiacol glutathionylpentosylhexoside	−	−	−	−	−	−	−	−	−	−	+
Fertaroyl guaiacol	+	+	+	+	+	−	+	+	+	+	+
Malonyl guaiacol	+	−	−	+	+	−	−	−	+	−	+
4-methyl guaiacol coumaroylpentoside	+	+	+	−	+	−	+	+	−	−	+
Caftaroyl 4-methyl guaiacol	−	−	−	−	−	−	−	−	+	−	+
Coutaroyl 4-methyl guaiacol	−	+	−	+	+	+	−	+	+	+	+
Tartaroyl 4-methyl guaiacol (isomer 1)	−	+	−	−	+	−	+	+	+	+	+
Tartaroyl 4-methyl guaiacol (isomer 2)	−	−	−	+	+	+	+	−	−	−	+
4-methyl syringol fertaroylhexoside	+	+	−	−	+	−	+	−	+	+	+
4-methyl syringol hexuronide (isomer 1)	+	+	+	−	+	+	+	+	+	+	+
4-methyl syringol hexuronide (isomer 2)	+	−	+	−	+	−	−	+	+	+	+
Sinapoyl syringol/vanillyl alcohol	+	+	+	−	+	+	+	+	+	+	+

Compounds	Potentially smoke−related glycosides										
4-ethyl syringol hexuronide	+	+	+	+	+	−	+	+	+	+	+
Sinapoyl alcohol caftaroyldihexoside	−	−	−	−	−	−	−	−	−	−	+
Sinapoyl alcohol hexoside	−	−	−	−	−	−	+	+	+	+	+
Caffeoyl syringyl alcohol	+	+	−	+	−	−	−	+	+	+	+
Coumaroyl syringyl alcohol	−	+	−	+	−	+	−	−	−	+	+
Syringyl alcohol caffeoylhexoside	−	−	−	−	−	−	−	−	−	−	+
Syringyl alcohol coumaroylhexoside (isomer 1)	−	−	−	−	−	−	−	−	−	−	+
Syringyl alcohol coumaroylhexoside (isomer 2)	−	−	−	−	−	−	−	−	−	−	+
Ethyl vanillin galloylhexoside	+	+	+	+	−	+	−	+	−	+	+
Ethyl vanillin hexuronide	−	+	+	−	+	−	+	+	+	+	+
Vanillyl galloylhexoside	−	−	−	−	+	+	−	+	+	−	+
malyl 4-vinylguaiacol	−	−	−	−	−	−	−	−	−	−	+
4-vinylguaiacol feruloylpentosylhexoside	−	−	−	−	−	−	−	−	−	−	+
4-vinylguaiacol galloylpentoside	+	+	+	+	+	+	+	+	−	+	+
Malyl 4-vinyl phenol	+	+	+	−	+	−	−	+	−	+	+

Detection of smoke-taint-related glycosides following fermentation with 10 different S. *cerevisiae* strains compared to a “No yeast” control. The presence of a compound is indicated by “**+**,” whereas “−” denotes hydrolysis or absence. Compounds are grouped into smoke-taint glycosides (commonly recognized smoke-taint glycosides; upper section) and potentially smoke-related glycosides (rarely observed in wine but derived from the same source; lower section). This table illustrates strain-specific differences in the metabolism of glycosidically bound phenols, highlighting the potential impact of yeast selection on smoke-taint release during fermentation.

A detailed analysis of glycoside hydrolysis profiles revealed clear strain-specific preferences in substrate utilization. In total, 31 glycoside compounds, comprising 16 commonly recognized smoke-taint compounds and 15 potentially smoke-related glycosides, were identified as being hydrolyzed by yeasts (Table 1). Of these, 8 compounds, including *guaiacol galloyldipentoside*, *guaiacol glutathionylpentosylhexoside*, *4-vinylguaiacol feruloylpentosylhexoside*, *malyl 4-vinylguaiacol*, *sinapoyl alcohol caftaroyldihexoside*, and 2 isomers of *syringyl alcohol coumaroylhexoside*, were consistently hydrolyzed by all 10 yeast strains. Additionally, 8 of 31 compounds were hydrolyzed by more than half of the yeast strains tested, including *4-ethylguaiacol hexonate*, *caftaroyl 4-methyl guaiacol*, *tartaroyl 4-methyl guaiacol* (isomer 2), *guaiacol caftaroylpentoside*, *sinapoyl alcohol hexoside*, *coumaroyl syringyl alcohol*, and *vanillyl galloylhexoside*. These results suggest that most of the *S. cerevisiae* strains express the enzymes necessary to hydrolyze these substrates under wine fermentation conditions. In contrast, some compounds were hydrolyzed exclusively by specific strains. For example, *4-ethyl syringol hexuronide* and *fertaroyl guaiacol* were hydrolyzed only by UCD525, while 4-methyl *syringol hexuronide* (isomer 1) and *sinapoyl syringol/vanillyl alcohol* were hydrolyzed solely by UCD514. These results indicate that certain yeast strains possess a broader substrate range or higher enzymatic efficiency for releasing smoke-taint-active compounds from glycosidically-bound precursors. Such strain-specific hydrolysis highlights the potential for targeted application of selected *S. cerevisiae* strains to mitigate smoke-taint precursors in wine.

It is important to note that the use of a filtered flavor extract in the mini-scale fermentation limited the overall number of detectable compounds, thereby reducing the pool of analyzable glycosides. Despite this limitation, the observed differences in hydrolysis among the *S. cerevisiae* strains indicate varying degrees of enzyme-substrate specificity, reinforcing the strain-dependent nature of glycosidase activity in wine fermentation.

### Glycoside hydrolysis by malolactic bacteria

For the malolactic fermentation, 54 samples were analyzed, representing 5 bacterial strains and one control without added bacteria. Each fermentation was conducted in triplicate, and each biological replicate was further prepared in three technical repeats. Qualitative and quantitative analyses were conducted using the LC–MS data obtained from the mini-scale malolactic fermentations. Across all samples, approximately 2,650 compounds were detected.

**Figure 3:**
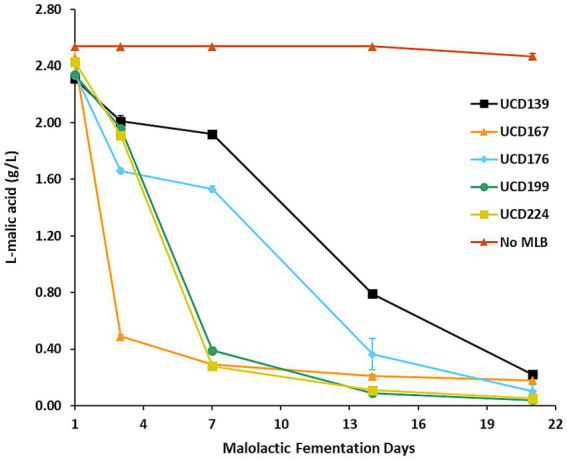
Malolactic fermentation in the synthetic wine. L-malic acid concentrations were measured over 21 days during malolactic fermentation carried out by 5 different *O. oeni* strains, with a “No MLB” (no bacteria) control included for comparison. Each data point represents the mean of 3 independent biological replicates, and error bars indicate standard deviations.

A data mining approach similar to that applied in the yeast fermentations was used, employing the same in-house database of glycoside accurate masses, which identified 29 glycoside compounds. These 29 glycosides, which were consistently retained in the “No MLB” control and therefore served as a baseline reference, were used to evaluate enzymatic hydrolysis across all bacterial strains (Supplementary Table S6). Of these, 5 compounds were not hydrolyzed by any of the *O. oeni* strains, indicating resistance to both enzymatic and chemical hydrolysis. Among the 5 *O. oeni* strains tested, UCD199 exhibited the highest glycosidase activity, hydrolyzing 16 of 29 compounds, whereas UCD224 presented the lowest activity, hydrolyzing only 6 (Supplementary Table S5). Interestingly, UCD199 and UCD224 showed comparable malolactic fermentation kinetics (Figure 3), suggesting that factors beyond overall metabolic activity, such as stress tolerance and genetic variation, may contribute to the observed differences in glycosidase activity.

In total, 24 glycoside compounds were identified as hydrolyzed by one or more bacterial strains, including 7 commonly recognized smoke-taint-associated compounds and 17 potentially smoke-related glycosides (Table 2). Notably, *guaiacol tartaroylpentoside* was hydrolyzed by all 5 bacterial strains, indicating a common enzymatic substrate. 10 of the 24 compounds were hydrolyzed by more than half of the tested *O. oeni* strains, including *4-vinyl catechol galloylhexoside*, *4-vinyl phenol coumaroylhexoside*, *coniferaldehyde malylpentoside*, *coniferol/4-vinyl syringol feruloyl*, *cresol coumaroyldipentoside*, *guaiacol tartaroylpentoside*, *sinapoyl alcohol sinapoylhexoside*, *syringol/vanillyl alcohol galloyldipentoside*, *syringyl alcohol tartaroyldihexoside*, *syringyl alcohol caftaroylpentoside* (isomer 2). In contrast, several compounds showed clear strain-specific hydrolysis: *cresol caftaroylhexoside* and *sinapoyl alcohol tartaroylpentoside* (isomer 1) were hydrolyzed only by UCD199, while *sinapoyl alcohol tartaroylpentoside* (isomer 2) was hydrolyzed exclusively by UCD176. Among the strains, UCD199 demonstrated the strongest activity toward common smoke-taint-associated glycosides, whereas UCD139 showed a broader hydrolytic range targeting potentially smoke-related glycosides (Table 2).

**Table 2:** Hydrolysis of smoke-taint-related glycosides by bacterial strains.

	UCD224	UCD167	UCD176	UCD139	UCD199	No MLB
Compounds	Smoke-taint glycosides					
4-ethylguaiacol sinapoylhexoside	+	−	−	+	+	+
4-methyl guaiacol galloylhexoside	+	+	+	−	−	+
4-methyl syringol sinapoylhexoside	+	+	+	−	−	+
Cresol caftaroylhexoside	+	+	+	+	−	+
Cresol coumaroyldipentoside	−	−	−	+	−	+
Guaiacol tartaroylpentoside (isomer 1)	−	−	−	+	−	+
Guaiacol tartaroylpentoside (isomer 2)	−	−	−	−	−	+

Compounds	Potentially smoke-related glycosides					
Glutathionyl 4-ethylphenol	+	+	+	−	−	+
Coutaroyl 4-vinyl catechol	+	−	−	+	+	+
4-vinyl catechol galloylhexoside	+	−	−	−	−	+
4-vinyl phenol coumaroylhexoside	−	+	+	−	−	+
Coniferaldehyde malylpentoside	+	−	−	−	+	+
Feruloyl coniferol/4-vinyl syringol	−	+	+	−	−	+
Sinapaldehyde tartaroylpentoside	+	−	−	+	+	+
Sinapoyl alcohol caffeoylpentoside	+	+	+	−	−	+
Sinapoyl alcohol maloylpentosylhexoside	+	+	+	−	−	+
Sinapoyl alcohol sinapoylhexoside	+	−	−	−	−	+
Sinapoyl alcohol tartaroylpentoside (isomer 1)	+	+	+	+	−	+
Sinapoyl alcohol tartaroylpentoside (isomer 2)	+	+	−	+	+	+
Syringol/vanillyl alcohol galloyldipentoside	−	+	+	−	−	+
Syringyl alcohol tartaroyldihexoside	+	−	−	−	−	+
Syringyl alcohol caftaroylpentoside (isomer 1)	+	−	−	+	+	+
Syringyl alcohol caftaroylpentoside (isomer 2)	+	−	−	−	+	+
Syringyl alcohol coutaroylhexoside	+	−	−	+	+	+

Hydrolytic activity of 5 *O. oeni* strains on smoke-taint-related glycosides, compared to a “No MLB” (no bacteria) control. The presence of a compound following malolactic fermentation is indicated by “**+**,” while “−” denotes hydrolysis or absence. Compounds are grouped into smoke-taint glycosides (commonly recognized smoke-taint glycosides; upper section) and potentially smoke-related glycosides (rarely observed in wine but derived from the same source; lower section). This table demonstrates strain-dependent variability in glycosidase activity, reflecting differences in the capacity of malolactic bacteria to liberate volatile phenols from bound precursors.

Although only a limited number of glycosides were detected under mini-scale malolactic fermentation conditions, these findings suggest that glycosidase activity in *O. oeni* is both strain-dependent and compound-specific, with potential implications for the targeted modulation of smoke-taint precursors during malolactic fermentation.

### Principal component analysis

To evaluate the impact of yeast strains on glycosidic compounds, principal component analysis (PCA) was conducted using glycoside data derived from mini-scale alcoholic fermentations (Figure 2). The “No yeast” control formed a clearly separated cluster, confirming the significant enzymatic contribution of yeast strains to the modification of glycosidic compounds. PCA revealed partial overlap among clusters of different yeast strains, suggesting shared glycosidase hydrolysis activity, which is consistent with the ~ 25% of common substrates detected across all strains (8 out of 31) (Table 1). However, distinct separations were also observed, such as between UCD506 and UCD2784, indicating strain-specific differences in glycosidase hydrolysis. Together, the PCA results and glycoside hydrolysis profiles demonstrate that *S. cerevisiae* strains show both shared and strain-specific effects on glycoside hydrolysis during alcoholic fermentation.

**Figure 4:**
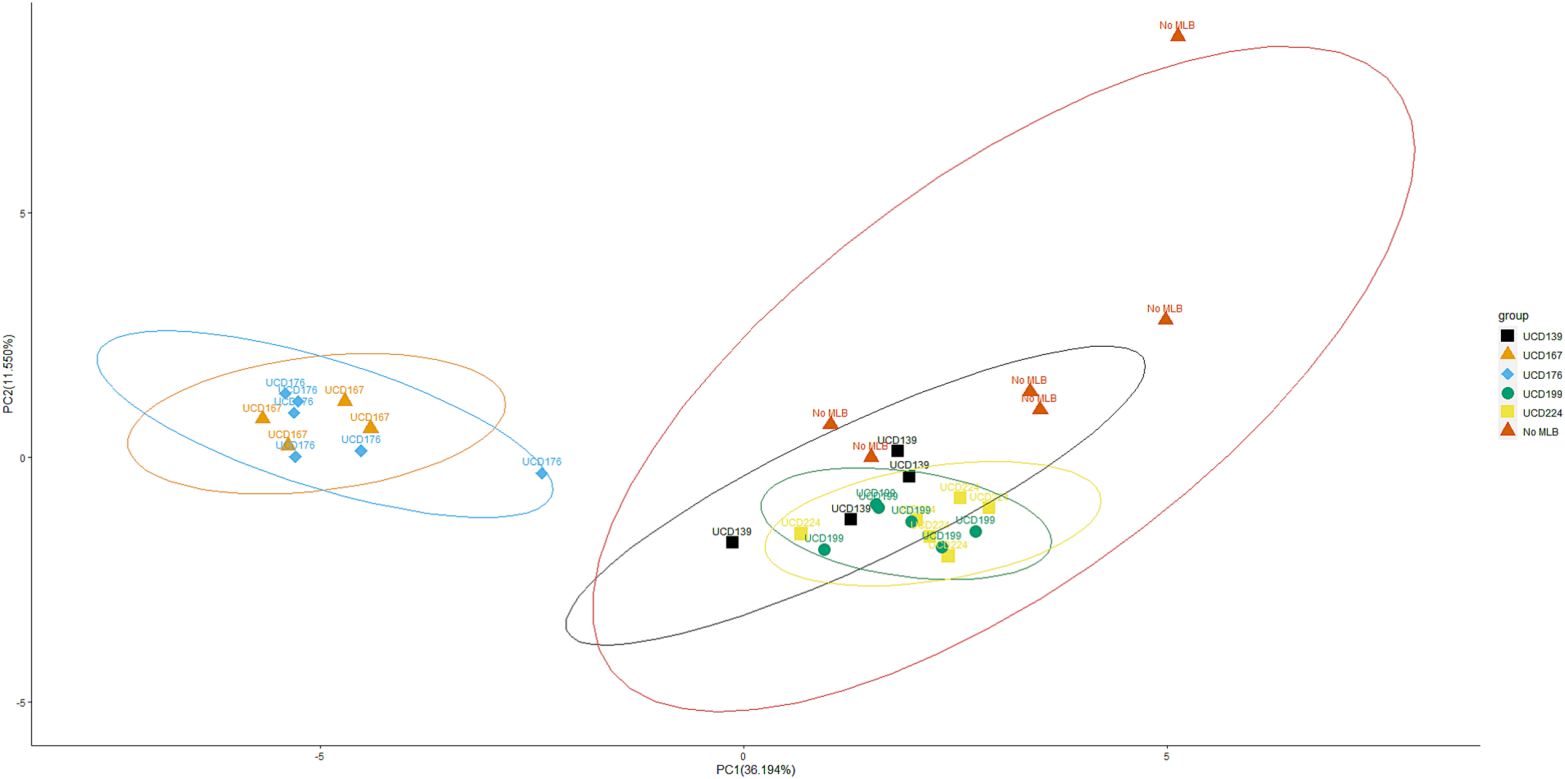
Principal component analysis (PCA) of glycosides after malolactic fermentation. PCA plot showing the distribution of glycoside profiles obtained after malolactic fermentation with 5 different *O. oeni* strains, compared to a “No MLB” (no bacteria) control. Each point represents both technical and biological replicates. For each strain, 9 samples were analyzed in total: 3 independent biological replicates, each prepared in triplicate as technical repeats, to minimize variability from HPLC instrument runs. Datasets producing inconsistent results were excluded, and only “clean” data were retained for PCA analysis. Data are projected onto the first two principal components, with the percentage of total variance explained by each component indicated on the respective axes. Ellipses represent the 95% confidence interval around the multivariate mean of each strain, providing a visual estimate of clustering and variability.

Similarly, PCA was performed on glycoside profiles obtained from malolactic fermentations with 5 *O. oeni* strains and a non-inoculated control (“No MLB”) (Figure 4). The analysis revealed a clear separation of UCD167 and UCD176 from the remaining *O. oeni* strains and the control, highlighting differences in glycosidase activity and their respective capacities to alter glycosidic composition during malolactic fermentation. Although the control (“No MLB”) and three *O. oeni* strains (UCD 139, UCD 199, and UCD224) clustered closely, each strain exhibited distinct glycoside preference (Table 2), suggesting strain-specific enzymatic hydrolysis of smoke-taint-related glycosides.

## Discussion

Glycosidically bound volatile aroma compounds serve as an important reservoir of potential aroma in wines, and their hydrolysis, whether mediated by microbial or chemical processes, significantly influences the final aromatic profile (Hjelmeland and Ebeler, 2015). In this study, we evaluated 10 *S. cerevisiae* and 5 *O. oeni* strains from the microbial culture collection at the University of California, Davis, to identify microbial candidates capable of mitigating undesirable smoke-taint compounds. LC–MS-based glycoside profiling enabled a comprehensive evaluation of enzymatic interactions between microbes and glycosidic precursors of volatile aromas. Our findings are consistent with previous reports and expand the understanding of the metabolic impact of wine yeast and bacteria on flavor precursors. The results implicate glycosidically bound volatiles as significant contributors to the sensory properties of wine, consistent with previous reports on fermented grape fruit and hop cone products (Caffrey and Ebeler, 2021). Overall, this work advances strategies for microbiological interventions aimed at improving wine quality by selectively promoting the release of desired aroma compounds while limiting those with a negative sensory impact, such as smoke-taint.

Interestingly, certain yeast strains, such as UCD522 and UCD557, showed delayed sugar utilization, while UCD2784 exhibited incomplete fermentation (Figure 1). These outcomes likely reflect strain-specific variability in traits such as stress tolerance, nitrogen requirements, and the ability to ferment under high sugar or ethanol concentration (Berthels et al., 2004). The lowered glycosidase activity observed in strain UCD522 (Table 1; Supplementary Table S3) may therefore be linked to inherent strain differences or its slower fermentation kinetics. Additionally, *caftaroyl 4-methyl guaiacol* was detected in UCD2784 (Table 1), whereas all other yeast strains were able to hydrolyze this compound. This result may be explained by the incomplete fermentation of UCD2784, which likely limited caftaroyl esterase activity, an essential step preceding *β*-glucosidase-mediated hydrolysis of the glycoside.

Moreover, glycosidase enzymes in *S. cerevisiae* are subjected to glucose repression, whereby their activity is inhibited in the presence of abundant free glucose. Under such conditions, there is little metabolic incentive for the yeast to release glucose from glycosidic precursors. Consequently, strains with delayed sugar consumption (e.g., UCD522) (Figure 1) may exhibit slower or incomplete precursor hydrolysis (Supplementary Table S3). This mechanism may also explain why, in certain strains (e.g., UCD525), efficient glucose depletion (Figure 1) coincided with increased release of smoke-taint glycosides (Supplementary Table S3), emphasizing the interplay between sugar availability and enzymatic aroma liberation. Collectively, these findings highlight the need for further investigation into strain-specific stress responses and metabolic adaptability, particularly regarding glycosidase expression profiles under varying environmental conditions such as sugar and nitrogen availability (Roca-Mesa et al., 2020).

Our findings highlight the pivotal role of yeast and malolactic bacteria in modulating glycosidic compounds in wine, suggesting a biologically driven strategy to mitigate smoke-taint in wines produced from wildfire-exposed grapes. Notably, among the yeasts, strains UCD514 and UCD525 hydrolyzed the greatest number of glycoside compounds (21 out of 93 observed, Supplementary Table S3), while among the bacteria, strain UCD199 hydrolyzed the highest number (16 out of 29 observed, Supplementary Table S5). The robust hydrolytic activity of these strains emphasizes their potential for targeted application in precision fermentation approaches. These results broaden previous research demonstrating strain-specific variability in *β*-glucosidase activity and its impact on volatile aroma release (Whitmore et al., 2021). Moreover, yeast and bacterial glycosidases exhibit substrate specificity. In this study, two glycosides, *4-methyl guaiacol galloylhexoside* and *4-vinyl catechol galloylhexoside*, were detected during both yeast alcoholic fermentation and bacterial malolactic fermentation (Supplementary Tables S4, S6). Neither glycoside was hydrolyzed by any tested *S. cerevisiae* strains, whereas both were hydrolyzed by most of the tested *O. oeni* strains (Table 2). These findings emphasize that, in wine studies, the contributions of both yeast and bacteria must be considered when evaluating flavor evolution.

The consistent hydrolysis of certain glycosides across all *S. cerevisiae* strains (i.e., *guaiacol galloyldipentoside, guaiacol glutathionylpentosylhexoside, 4-vinylguaiacol feruloylpentosylhexoside, malyl 4-vinylguaiacol, sinapoyl alcohol caftaroyldihexoside, and two isomers of syringyl alcohol coumaroylhexoside*) or across all *O. oeni* strains (i.e., *guaiacol tartaroylpentoside*) suggests the presence of conserved enzymatic mechanisms. In contrast, variability in the selective hydrolysis of other compounds points to the influence of microbial genotype on substrate specificity. This dual pattern of conserved and strain-dependent enzymatic activity has also been reported in previous studies involving wine-related yeast species (Belda et al., 2016). These results align with earlier findings indicating that enzymatic cleavage of glycosidic bonds plays a critical role in the release of bound volatile aroma compounds (Rodriguez-Nogales et al., 2024).

While this study focused on *S. cerevisiae* and *O. oeni*, it is important to note that non-*Saccharomyces* yeasts such as *Metschnikowia* and *Hanseniaspora* often exhibit strong extracellular *β*-glucosidase activities and have been proposed as tools for enhancing aroma release during wine fermentations (Belda et al., 2016). Utilizing non-*Saccharomyces* yeasts in future studies under winemaking conditions would allow direct comparisons of their glycosidase activity with that of *Saccharomyces*, thereby broadening the scope of aroma modulation strategies.

Principal component analysis (PCA) further confirmed that both yeast and bacterial treatments significantly altered the glycosidic composition relative to the controls (Figures 2, 4). The PCA results highlight distinct glycoside hydrolysis patterns among *O. oeni* strains, with UCD167 and UCD176 forming clusters clearly separated from other strains (Figure 4), suggesting strain-specific potential to modulate smoke-taint precursor metabolism. In contrast, the *S. cerevisiae* strains exhibited substantial overlap, reflecting broadly similar glycosidase activity profiles under the tested conditions. Notably, the PCA was performed exclusively on putative smoke-taint-related glycosides, corresponding to the compound set presented in Tables 1, 2 and detailed in Supplementary Tables S4, S6. This focused approach enables a clear interpretation of both general and strain-dependent methods driving the release of undesirable aroma precursors, providing insight into how targeted strain selection could be employed to better control such aroma outcomes. Collectively, these findings emphasize the potential of leveraging microbial diversity to shape wine aroma profiles in desired directions (Malicanin et al., 2020).

To apply the observed microbial glycosidase activities to practical winemaking, future work should focus on developing strategies that mitigate the release of undesirable aroma volatiles while enhancing the liberation of favorable ones. One promising approach involves selecting microbial strains with glycosidase enzymes that exhibit greater substrate specificity toward desirable glycosides. Additionally, optimizing inoculation timing, through sequential or co-inoculation fermentation, may allow for more targeted hydrolysis of glycosidic precursors. Finally, post-fermentation separation techniques, such as adsorption using selective resins or membrane-based fractionation, represent viable strategies to selectively remove unwanted compounds without compromising beneficial aroma contributions (Fudge et al., 2011). Together, these approaches provide a technical framework for the controlled utilization of glycosidase activity to improve wine aromatic quality.

To further validate these findings under natural winemaking conditions, we conducted fermentation in 20 L and 120 L fermenters using both smoke-exposed and non-smoke-exposed grapes (manuscript in preparation). Importantly, future work should integrate enzymatic hydrolysis data with sensory analysis to determine whether observed hydrolytic activity translates into perceptible aromatic benefits. In parallel, examining how specific strains simultaneously enhance favorable aroma expression and cleave smoke-taint precursors will be essential for balancing taint reduction with aromatic enhancement. This dual-focus approach will provide valuable insights into both the risks and opportunities associated with microbial enzymatic activity in smoke-affected wines. Collectively, this work contributes to a growing body of evidence supporting microbial modulation of wine composition as a feasible strategy for mitigating smoke-taint effects while optimizing aromatic expression (Oberholster et al., 2022).

## Data Availability

The original contributions presented in the study are included in the article/Supplementary material, further inquiries can be directed to the corresponding authors.
