# Supplementary material for: Investigation of smoke-taint precursor modification by glycosidase activity in diverse wine yeast and bacterial strains
Source: Front Microbiol. 2025 Oct 31;16:1679638. doi: 10.3389/fmicb.2025.1679638 (PMC12616739; doi:10.3389/fmicb.2025.1679638)
Supplement: Supplementary file 1 [file Table_1.docx]

**Supplement Materials**

**Table S1. Yeast and malolactic bacterial strain information**

| **Strain Name** | **Genus** | **Species** | **Source** |  |
| --- | --- | --- | --- | --- |
| UCD506 | *Saccharomyces* | *cerevisiae* | Wine, Bordeaux | UC Davis |
| UCD509 | *Saccharomyces* | *cerevisiae* | Wine, Burgundy II | UC Davis |
| UCD512 | *Saccharomyces* | *cerevisiae* | Cognac fermentation | UC Davis |
| UCD514 | *Saccharomyces* | *cerevisiae* | Wine, Geisenheim | UC Davis |
| UCD522 | *Saccharomyces* | *cerevisiae* | Wine, Montrachet | UC Davis |
| UCD525 | *Saccharomyces* | *cerevisiae* | Wine, Port | UC Davis |
| UCD557 | *Saccharomyces* | *cerevisiae* | Wine, Sherry | UC Davis |
| UCD932 | *Saccharomyces* | *cerevisiae* | Grapes, vineyard, Italy | UC Davis |
| UCD2784 | *Saccharomyces* | *cerevisiae* | Grapes, vineyard, California | UC Davis |
| UCD2790 | *Saccharomyces* | *cerevisiae* | Wine yeast, AWRI 1631 | UC Davis |
| UCD139 | *Oenococcus* | *oeni* | Wine, Switzerland | UC Davis |
| UCD167 | *Oenococcus* | *oeni* | Wine, Pinot Noir | UC Davis |
| UCD176 | *Oenococcus* | *oeni* | Wine, Spain | UC Davis |
| UCD199 | *Oenococcus* | *oeni* | Wine, Merlot | UC Davis |
| UCD224 | *Oenococcus* | *oeni* | Wine, Chile | UC Davis |

**Table S2. Composition of synthetic grape juice and synthetic wine media used in this study**

|  | **Synthetic Juice** | **Synthetic Wine** |
| --- | --- | --- |
| Potassium Phosphate, monobasic | 2.00 g/L | 2.00 g/L |
| Magnesium Sulfate | 2.00 g/L | 2.00 g/L |
| Calcium Chloride | 0.20 g/L | 0.20 g/L |
| Amino Acids | 3.85 g/L | 3.85 g/L |
| Adenine | 50 mg/L | 50 mg/L |
| Arginine | 250 mg/L | 250 mg/L |
| Aspartic Acid | 400 mg/L | 400 mg/L |
| Histidine | 100 mg/L | 100 mg/L |
| Isoleucine | 250 mg/L | 250 mg/L |
| Leucine | 500 mg/L | 500 mg/L |
| Lysine | 250 mg/L | 250 mg/L |
| Methionine | 100 mg/L | 100 mg/L |
| Phenylalanine | 250 mg/L | 250 mg/L |
| Tryptophan | 250 mg/L | 250 mg/L |
| Threonine | 500 mg/L | 500 mg/L |
| Tyrosine | 250 mg/L | 250 mg/L |
| Valine | 700 mg/L | 700 mg/L |
| Trace elements |  |  |
| Manganese Sulfate | 0.80 mg/L | 0.80 mg/L |
| Zinc Sulfate | 0.80 mg/L | 0.80 mg/L |
| Ferric Chloride | 0.40 mg/L | 0.40 mg/L |
| Copper Sulfate | 0.08 mg/L | 0.08 mg/L |
| Boric Acid | 1.00 mg/L | 1.00 mg/L |
| Sodium Molybdate | 0.40 mg/L | 0.40 mg/L |
| Potassium Iodide | 0.20 mg/L | 0.20 mg/L |
| Vitamins |  |  |
| Inositol | 4.0 mg/L | 4.0 mg/L |
| Pyridoxine Hydrochloride | 0.8 mg/L | 0.8 mg/L |
| Niacin | 0.8 mg/L | 0.8 mg/L |
| Calcium Pantothenate | 0.8 mg/L | 0.8 mg/L |
| Thiamine Hydrochloride | 0.8 mg/L | 0.8 mg/L |
| P-Aminobenzoic Acid | 0.4 mg/L | 0.4 mg/L |
| Riboflavin | 0.4 mg/L | 0.4 mg/L |
| Biotin | 0.004 mg/L | 0.004 mg/L |
| Folic Acid | 0.004 mg/L | 0.004 mg/L |
| Sugar Added |  |  |
| Glucose | 100 g/L | 0 g/L |
| Fructose | 100 g/L | 0 g/L |
| Ethanol Added | No | 12% |
| Acid Added |  |  |
| Tartaric acid (g) | 3 g/L | 3 g/L |
| L-malic acid (g) | 2 g/L | 2/g/L |
| pH | 3.5 | 3.5 |
| Flavor Extract Added | 20% | 20% |

**Table S3. Total glycosides and those hydrolyzed by yeast strains**

|  | **UCD506** | **UCD509** | **UCD512** | **UCD514** | **UCD522** | **UCD525** | **UCD557** | **UCD932** | **UCD2784** | **UCD2790** | **No Yeast** |
| --- | --- | --- | --- | --- | --- | --- | --- | --- | --- | --- | --- |
| **Total Glycosides Observed** | 76 | 77 | 74 | 72 | 80 | 72 | 75 | 79 | 76 | 79 | 93 |
| **Glycosides Hydrolysis by Yeasts** | 17 | 16 | 19 | 21 | 13 | 21 | 18 | 14 | 17 | 14 | 0 |

**Table S4. Glycosides detected across yeast fermentation samples**

| **Compounds** | **UCD506** | **UCD509** | **UCD512** | **UCD514** | **UCD522** | **UCD525** | **UCD557** | **UCD932** | **UCD2784** | **UCD2790** | **No Yeast** |
| --- | --- | --- | --- | --- | --- | --- | --- | --- | --- | --- | --- |
| tartaroyl coniferaldehyde | + | + | + | + | + | + | + | + | + | + | + |
| cresol maloyldipentoside (isomer 1) | + | + | + | + | + | + | + | + | + | + | + |
| cresol maloyldipentoside (isomer 2) | + | + | + | + | + | + | + | + | + | + | + |
| cresol maloyldipentoside (isomer 3) | + | + | + | + | + | + | + | + | + | + | + |
| cresol maloyldipentoside (isomer 4) | + | + | + | + | + | + | + | + | + | + | + |
| 4-ethyl syringol hexuronide | + | + | + | + | + | − | + | + | + | + | + |
| 4-ethylguaiacol galloylpentoside | + | + | + | + | + | + | + | + | + | + | + |
| 4-ethylguaiacol hexonate | + | + | − | − | + | − | + | − | − | − | + |
| 4-ethylguaiacol hexosylhexoside | + | − | + | + | + | + | + | + | − | + | + |
| malonyl 4-ethylguaiacol | + | + | + | + | + | + | + | + | + | + | + |
| 4-ethylguaiacol hexuronide | + | + | + | + | + | + | + | + | + | + | + |
| 4-ethylguaiacol malonylpentoside | + | + | + | + | + | + | + | + | + | + | + |
| 4-ethylguaiacol pentosylpentoside (isomer 1) | + | + | + | + | + | + | + | + | + | + | + |
| 4-ethylguaiacol pentosylpentoside (isomer 2) | + | + | + | + | + | + | + | + | + | + | + |
| 4-ethylphenol hexuronide | + | + | + | + | + | + | + | + | + | + | + |
| acetyl guaiacol (isomer 1) | + | + | + | + | + | + | + | + | + | + | + |
| acetyl guaiacol (isomer 2) | + | + | + | + | + | + | + | + | + | + | + |
| caftaroyl guaiacol | + | + | + | + | + | + | + | + | + | + | + |
| fertaroyl guaiacol | + | + | + | + | + | − | + | + | + | + | + |
| guaiacol caffeoylhpentosylhexoside (isomer 1) | + | + | + | + | + | + | + | + | + | + | + |
| guaiacol caffeoylhpentosylhexoside (isomer 2) | + | + | + | + | + | + | + | + | + | + | + |
| guaiacol caftaroylpentoside (isomer 1) | + | + | + | + | + | + | + | + | + | + | + |
| guaiacol caftaroylpentoside (isomer 2) | − | − | + | − | + | + | − | + | − | + | + |
| guaiacol coumaroyldihexoside (isomer 1) | + | + | + | + | + | + | + | + | + | + | + |
| guaiacol coumaroyldihexoside (isomer 2) | + | + | + | + | + | + | + | + | + | + | + |
| guaiacol galloylpentoside | − | − | − | − | − | − | − | − | − | − | + |
| guaiacol glutathionylpentosylhexoside | − | − | − | − | − | − | − | − | − | − | + |
| guaiacol sinapoyldihexoside | + | + | + | + | + | + | + | + | + | + | + |
| guaiacol sinapoylhexoside | + | + | + | + | + | + | + | + | + | + | + |
| malonyl guaiacol (isomer 1) | + | + | + | + | + | + | + | + | + | + | + |
| malonyl guaiacol (isomer 2) | + | − | − | + | + | − | − | − | + | − | + |
| caftaroyl 4-methyl guaiacol | − | − | − | − | − | − | − | − | + | − | + |
| coutaroyl 4-methyl guaiacol (isomer 1) | + | + | + | + | + | + | + | + | + | + | + |
| coutaroyl 4-methyl guaiacol (isomer 2) | + | + | + | + | + | + | + | + | + | + | + |
| coutaroyl 4-methyl guaiacol (isomer 3) | − | + | − | + | + | + | − | + | + | + | + |
| 4-methyl guaiacol coumaroylpentoside | + | + | + | − | + | − | + | + | − | − | + |
| galloyl 4-methyl guaiacol | + | + | + | + | + | + | + | + | + | + | + |
| 4-methyl guaiacol galloylhexoside | + | + | + | + | + | + | + | + | + | + | + |
| 4-methyl guaiacol hexonate | + | + | + | + | + | + | + | + | + | + | + |
| 4-methyl guaiacol maloylhexoside | + | + | + | + | + | + | + | + | + | + | + |
| 4-methyl guaiacol pentosylpentoside (isomer 1) | + | + | + | + | + | + | + | + | + | + | + |
| 4-methyl guaiacol pentosylpentoside (isomer 2) | + | + | + | + | + | + | + | + | + | + | + |
| tartaroyl 4-methyl guaiacol (isomer 1) | − | + | − | − | + | − | + | + | + | + | + |
| tartaroyl 4-methyl guaiacol (isomer 2) | − | − | − | + | + | + | + | − | − | − | + |
| 4-methyl syringol acetylhexoside | + | + | + | + | + | + | + | + | + | + | + |
| 4-methyl syringol fertaroylhexoside | + | + | − | − | + | − | + | − | + | + | + |
| 4-methyl syringol hexosylhexoside | + | + | + | + | + | + | + | + | + | + | + |
| 4-methyl syringol hexuronide (isomer 1) | + | + | + | − | + | + | + | + | + | + | + |
| 4-methyl syringol hexuronide (isomer 2) | + | − | + | − | + | − | − | + | + | + | + |
| 4-vinyl catechol caffeoyldihexoside | + | + | + | + | + | + | + | + | + | + | + |
| 4-vinyl catechol galloyl | + | + | + | + | + | + | + | + | + | + | + |
| 4-vinyl catechol galloylhexoside (isomer 1) | + | + | + | + | + | + | + | + | + | + | + |
| 4-vinyl catechol galloylhexoside (isomer 2) | + | + | + | + | + | + | + | + | + | + | + |
| 4-vinyl catechol malyl | + | + | + | + | + | + | + | + | + | + | + |
| 4-vinyl catechol pentosylpentoside (isomer 1) | + | + | + | + | + | + | + | + | + | + | + |
| 4-vinyl catechol pentosylpentoside (isomer 2) | + | + | + | + | + | + | + | + | + | + | + |
| acetyl 4-vinylguaiacol | + | + | + | + | + | + | + | + | + | + | + |
| malyl 4-vinylguaiacol (isomer 1) | + | + | + | + | + | + | + | + | + | + | + |
| malyl 4-vinylguaiacol (isomer 2) | + | + | + | + | + | + | + | + | + | + | + |
| malyl 4-vinylguaiacol (isomer 2) | − | − | − | − | − | − | − | − | − | − | + |
| 4-vinylguaiacol acetylhexoside (isomer 1) | + | + | + | + | + | + | + | + | + | + | + |
| 4-vinylguaiacol acetylhexoside (isomer 2) | + | + | + | + | + | + | + | + | + | + | + |
| 4-vinylguaiacol feruloylpentosylhexoside | − | − | − | − | − | − | − | − | − | − | + |
| 4-vinylguaiacol galloylpentoside | + | + | + | + | + | + | + | + | − | + | + |
| 4-vinylguaiacol hexuronide | + | + | + | + | + | + | + | + | + | + | + |
| tartaroyl 4-vinylguaiacol (isomer 1) | + | + | + | + | + | + | + | + | + | + | + |
| tartaroyl 4-vinylguaiacol (isomer 2) | + | + | + | + | + | + | + | + | + | + | + |
| malyl 4-vinyl phenol (isomer 1) | + | + | + | + | + | + | + | + | + | + | + |
| malyl 4-vinyl phenol (isomer 2) | + | + | + | − | + | − | − | + | − | + | + |
| 4-vinyl phenol galloylhexoside | + | + | + | + | + | + | + | + | + | + | + |
| coniferol/4-vinyl syringol galloylhexoside | + | + | + | + | + | + | + | + | + | + | + |
| acetyl ethyl vanillin | + | + | + | + | + | + | + | + | + | + | + |
| ethyl vanillyl galloylhexoside | + | + | + | + | − | + | − | + | − | + | + |
| ethyl vanillyl hexuronide | − | + | + | − | + | − | + | + | + | + | + |
| tartaroyl sinapaldehyde | + | + | + | + | + | + | + | + | + | + | + |
| hexosyl sinapoyl alcohol | − | − | − | − | − | − | + | + | + | + | + |
| sinapoyl alcohol caftaroyldihexoside | − | − | − | − | − | − | − | − | − | − | + |
| syringaldehyde hexonate | + | + | + | + | + | + | + | + | + | + | + |
| caffeoyl syringyl alcohol | + | + | − | + | − | − | − | + | + | + | + |
| coumaroyl syringyl alcohol (isomer 1) | + | + | + | + | + | + | + | + | + | + | + |
| coumaroyl syringyl alcohol (isomer 2) | − | + | − | + | − | + | − | − | − | + | + |
| syringyl alcohol caffeoylhexoside | − | − | − | − | − | − | − | − | − | − | + |
| syringyl alcohol coumaroylhexoside (isomer 1) | − | − | − | − | − | − | − | − | − | − | + |
| syringyl alcohol coumaroylhexoside (isomer 2) | − | − | − | − | − | − | − | − | − | − | + |
| caftaroyl syringol/vanillyl alcohol (isomer 1) | + | + | + | + | + | + | + | + | + | + | + |
| caftaroyl syringol/vanillyl alcohol (isomer 2) | + | + | + | + | + | + | + | + | + | + | + |
| syringol/vanillyl alcohol hexuronide (isomer 1) | + | + | + | + | + | + | + | + | + | + | + |
| syringol/vanillyl alcohol hexuronide (isomer 2) | + | + | + | + | + | + | + | + | + | + | + |
| sinapoyl syringol/vanillyl alcohol | + | + | + | − | + | + | + | + | + | + | + |
| vanillyl galloylhexoside (isomer 1) | + | + | + | + | + | + | + | + | + | + | + |
| vanillyl galloylhexoside (isomer 1) | + | + | + | + | + | + | + | + | + | + | + |
| vanillyl galloylhexoside (isomer 1) | + | + | + | + | + | + | + | + | + | + | + |
| vanillyl galloylhexoside (isomer 1) | − | − | − | − | + | + | − | + | + | − | + |

Presence (**+**) or hydrolysis (−) of glycosides by each yeast strain.

**Table S5. Total glycosides and those hydrolyzed by bacterial strains**

|  | **UCD224** | **UCD167** | **UCD176** | **UCD139** | **UCD199** | **No MLB** |
| --- | --- | --- | --- | --- | --- | --- |
| **Total Glycosides Observed in all Samples** | 23 | 16 | 15 | 15 | 13 | 29 |
| **Glycosides Hydrolysis by Bacteria** | 6 | 13 | 14 | 14 | 16 | 0 |

**Table S6. Glycosides detected in bacterial malolactic fermentation samples**

| **Compounds** | **UCD224** | **UCD167** | **UCD176** | **UCD139** | **UCD199** | **No MLB** |
| --- | --- | --- | --- | --- | --- | --- |
| coniferaldehyde malonylpentoside | + | + | + | + | + | + |
| coniferaldehyde malylpentoside | + | − | − | − | + | + |
| coniferaldehyde tartaroylpentosylhexoside | + | + | + | + | + | + |
| feruloyl coniferol/4−vinyl syringol | − | + | + | − | − | + |
| cresol caftaroylhexoside | + | + | + | + | − | + |
| cresol coumaroyldipentoside | − | − | − | + | − | + |
| cresol galloylpentoside | + | + | + | + | + | + |
| 4−ethylguaiacol sinapoylhexoside | + | − | − | + | + | + |
| glutathionyl 4−ethylphenol | + | + | + | − | − | + |
| 4−methyl guaiacol galloylhexoside | + | + | + | − | − | + |
| 4−methyl syringol sinapoylhexoside | + | + | + | − | − | + |
| guaiacol tartaroylpentoside (isomer 1) | + | + | + | + | + | + |
| guaiacol tartaroylpentoside (isomer 2) | + | + | + | + | + | + |
| guaiacol tartaroylpentoside (isomer 3) | − | − | − | + | − | + |
| guaiacol tartaroylpentoside (isomer 4) | − | − | − | − | − | + |
| sinapaldehyde tartaroylpentoside | + | − | − | + | + | + |
| sinapoyl alcohol caffeoylpentoside | + | + | + | − | − | + |
| sinapoyl alcohol maloylpentosylhexoside | + | + | + | − | − | + |
| sinapoyl alcohol sinapoylhexoside | + | − | − | − | − | + |
| sinapoyl alcohol tartaroylpentoside (isomer 1) | + | + | + | + | − | + |
| sinapoyl alcohol tartaroylpentoside (isomer 2) | + | + | − | + | + | + |
| syringol/vanillyl alcohol galloyldipentoside | − | + | + | − | − | + |
| syringyl alcohol tartaroyldihexoside | + | − | − | − | − | + |
| syringyl alcohol caftaroylpentoside (isomer 1) | + | − | − | + | + | + |
| syringyl alcohol caftaroylpentoside (isomer 2) | + | − | − | − | + | + |
| syringyl alcohol coutaroylhexoside | + | − | − | + | + | + |
| coutaroyl 4−vinyl catechol | + | − | − | + | + | + |
| 4−vinyl catechol galloylhexoside | + | − | − | − | − | + |
| 4−vinyl phenol coumaroylhexoside | − | + | + | − | − | + |

Presence (**+**) or hydrolysis (−) of glycosides by each bacterial strain.
